# Hyponatremia in patients with severe anorexia nervosa was associated with more severe and longer duration of disease

**DOI:** 10.3389/fpsyt.2026.1835483

**Published:** 2026-05-29

**Authors:** Jeanie Meincke Egedal, Magnus Sjögren, Georgios Paslakis, René Klinkby Støving

**Affiliations:** 1 Center for Eating Disorders, Odense University Hospital & Mental Health Services in the Region of Southern Denmark, Odense, Denmark; 2 Endocrine Research Unit, Odense University Hospital & Clinical Institute, University of Southern Denmark, Vejle, Denmark; 3 Institute for Clinical Sciences, Umea University, Umea, Sweden; 4 Ruhr-University of Bochum, Medical Faculty, University Clinic for Psychosomatic Medicine and Psychotherapy, Lübbecke, Germany

**Keywords:** anorexia nervosa, disease severity, eating disorders, electrolyte imbalance, hyponatremia, psychopathology

## Abstract

**Introduction:**

Several mechanisms are thought to contribute to hyponatremia in patients with anorexia nervosa (AN). The aims of this descriptive, cross-sectional study among patients admitted to a specialized somatic unit for eating disorders (ED) were to determine the frequency of hyponatremia and to compare medical findings between patients with normonatremia and hyponatremia.

**Methods:**

This retrospective, descriptive cross-sectional study included patients admitted to the unit between December 2016 and October 2021. Demographic, medical history, and clinical data were extracted from medical records. Patients were categorized according to plasma sodium concentration (<135 mmol/L vs. ≥135 mmol/L).

**Results:**

Among 131 patients, 17 (13%) had hyponatremia at admission. Hyponatremia was associated with lower BMI, lower nadir BMI, longer disease duration, and an adverse biochemical profile (lower albumin, higher creatinine, higher platelet counts, and higher bicarbonate levels). Thirteen patients (10%) were deceased at follow-up; hyponatremia was associated with mortality in unadjusted analysis (OR 8.03, 95% CI 2.29–28.16) but not after multivariable adjustment.

**Discussion:**

The study found that 13% of patients admitted to a specialized somatic unit for ED had hyponatremia, which clustered with indicators of more severe and longstanding AN (lower BMI, lower nadir BMI, longer disease duration). Hyponatremia was associated with mortality in unadjusted analyses, but this association was attenuated after adjustment for age and illness severity. Despite the established potential of purging to induce hyponatremia, our findings suggest that, in this severely ill inpatient population, overall illness severity and chronic medical deterioration may be more important determinants of both hyponatremia and mortality risk than purging per se.

## 1 Introduction

Hyponatremia is a common electrolyte disturbance in hospitalized patients which may cause safety issues both during its development and correction (1). For example, acute and severe hyponatremia may cause brain edema resulting in confusion, seizures, and coma. In addition, correcting chronically low levels of sodium too fast may lead to central pontine myelinolysis, which is why the correction of hyponatremia should be done with caution (2).

Hyponatremia has been proven to be a strong independent prognostic marker for morbidity and mortality in many different populations, e.g., patients with heart failure, myocardial infarction, liver cirrhosis, renal insufficiency, diabetes, stroke, and bone fractures (3–9). Additionally, recent studies have shown a significant association between hyponatremia and reduced bone mineral density in patients with AN (10). In a study of 2960 patients consecutively admitted with a broad spectrum of medical and surgical diagnoses, hyponatremia was found to be strongly associated with increased all-cause mortality and longer admission length independently of diagnosis and clinical variables (11).

Anorexia nervosa (AN) is a condition with three diagnostic criteria according to The Diagnostic and Statistical Manual of Mental Disorders, Fifth Edition (DSM-5): i) Restriction of energy intake relative to requirements leading to a significantly low body weight in the context of age, sex, developmental trajectory, and physical health, ii) Intense fear of gaining weight or becoming fat, even though at underweight and iii) Disturbance in the way in which one’s body weight or shape is experienced, or denial of the seriousness of the current low body weight (12). There are two subtypes of AN: i) restrictive, and ii) binge-eating/purging. Purging is characterized by self-induced vomiting or misuse of diuretics, laxatives, or enemas. The severity of AN according to DSM-5 is based on body mass index (BMI). Nadir BMI is the lowest BMI prior to, or at admission. It has been found that nadir BMI is strongly inversely associated with mortality (13).

In patients with AN, there may be several different pathogenetic mechanisms concurrently contributing to hyponatremia. Patients with AN may engage in water loading to suppress appetite, rapidly gain temporary weight, or cleanse the body (14). Hypo-osmolar hyponatremia occurs when the oral consumption of solution-free fluid exceeds the renal capacity to excrete water (14). However, the frequency of water loading (or psychogenic polydipsia) in general in hospitalized psychiatric patients has been estimated to be as low as 5% (15). Hypotonic dehydration is another cause of hyponatremia. Following a restricted diet and low sodium intake, the renal excretion of sodium may be greater than the input, thus, resulting in hyponatremia (14). The syndrome of inappropriate antidiuretic hormone (SIADH) has also been reported in patients with AN (16). It is characterized by hypersecretion of antidiuretic hormone from the posterior pituitary gland and increased total water retention, again leading to hyponatremia. In addition, selective serotonin reuptake inhibitors (SSRI) are a well-recognized cause of hyponatremia, typically mediated by SIADH (17). Nevertheless, SSRI are frequently prescribed to patients with AN despite limited evidence of benefit (18). A condition related to the SIADH is the reset osmostat syndrome (ROS), a state in which the threshold for antidiuretic hormone secretion is lowered and which may also be present in AN (19, 20). ROS consists of a change in the normal plasma osmolality threshold, inducing chronic dysnatremia resistant to conventional treatments.

Hypokalemia is another common electrolyte disturbance in patients with AN associated with low plasma sodium levels, noticeably seen in individuals engaging in purging behavior (14, 21, 22). Misuse of laxatives increases the loss of potassium, while vomiting leads to loss of hydrogen ions and compensatory increased renal bicarbonate and potassium secretion. Most diuretics cause both hypokalemia and hyponatremia (23).

AN is the psychiatric disorder with the highest mortality among adolescents, and the second highest mortality among all ages (24). Patients with AN that are malnourished may thus need somatic treatment. The Nutrition Clinic is a somatic department specialized in treating severe cases of AN. This study was conducted to investigate the associations between hyponatremia and AN-related features and outcomes. The aims of this retrospective, descriptive cross-sectional study among patients admitted between the years 2016 and 2021 to a Danish specialized somatic unit for eating disorders (ED) were

- To determine the frequency of hyponatremia at admission.
- To compare AN with hyponatremia to that with normonatremia regarding BMI at admission, nadir BMI, age, the subtype of AN, medications, psychiatric comorbidities, mortality and other laboratory values.

## 2 Materials and methods

### 2.1 Study design, settings, and participants

Patients included in this study were admitted to a highly specialized medical nutrition unit for patients with severe ED in Denmark from December 31, 2016, to October 31, 2021. The unit is located at Odense University Hospital, Denmark. A total of 159 patients were identified and enrolled in this retrospective, descriptive cross-sectional study. Part of the cohort have participated in previous studies described elsewhere (25, 26). The diagnosis of AN was made by specialist psychiatrists. Patients with multiple admissions were only included once with their first hospital stay. Patients not diagnosed with AN were excluded from the study as well as those with missing data. Clinical data were collected from medical records at admission. Laboratory data were taken from the emergency department if the patient had been admitted there before transfer to the specialized ED unit. A total of 28 patients were excluded, 25 for not being diagnosed with AN and 3 due to missing/insufficient data leaving 131 patients for statistical analyses.

### 2.2 Study variables

The following clinical parameters were collected: weight (kg), height (m), BMI, nadir BMI, age (years), the subtype of AN (restricted/binge-purge), disease duration (years), length of stay (days), psychiatric comorbidities (yes/no) and if the patient were deceased at the time of data collection.

Information on the following prescribed drug use was collected: Hormonal contraceptives, SSRI, antipsychotics, and benzodiazepines. The following laboratory values were extracted: plasma (P)-hemoglobin, P-albumin, P-potassium, P-creatinine, P-Sodium, P-bicarbonate, P-leukocytes, P-platelets. P-Sodium at discharge was also documented.

### 2.3 Statistical methods

The study population was divided into groups based on their P-Sodium levels. A cut off point at <135 mmol/L was set to define hyponatremia based on the European Clinical Practice Guideline (23). Data were analyzed with Microsoft Excel (version 16.67) and Python code. Continuous data were visualized by QQ-plots to determine distribution. Student’s t-tests were used to calculate P-values for normally distributed data. Binomial data were analyzed with either Fisher’s exact test or Chi2-test, as appropriate. The distribution of BMI for the two groups was displayed in a bar chart. In additional exploratory analyses, patients were further stratified according to the severity of hyponatremia at admission into three categories: normal sodium (≥135 mmol/L), mild hyponatremia (130–134 mmol/L), and more severe hyponatremia (<130 mmol/L). Continuous variables across sodium categories were compared using one-way analysis of variance (ANOVA), while categorical variables were compared using Fisher’s exact test, as appropriate. Due to the limited number of patients with sodium concentrations <135 mmol/L, these analyses were considered exploratory. To examine the association between hyponatremia at admission and subsequent mortality, logistic regression analyses were performed. First, an unadjusted model was estimated with mortality (yes/no) as the dependent variable and hyponatremia (P-Sodium <135 mmol/L vs. ≥135 mmol/L) as the independent variable. Thereafter, a multivariable logistic regression model was constructed adjusting *a priori* for age, BMI at admission, disease duration, AN subtype, and sex. Odds ratios (OR) with 95% confidence intervals (CI) were reported. Robust standard errors were applied. Predicted probabilities of death according to sodium status were estimated using marginal effects. Statistical significance was defined as a two-sided p-value <0.05.

Statistical analyses were performed using Stata/BE version 19 (StataCorp LLC, College Station, TX, USA).

### 2.4 Ethics

This study was conducted in accordance with the Declaration of Helsinki. Permission to access past medical files was granted by the Department for Data and Personal Data Act in the Region of Southern Denmark (file no 21/61099). All data were handled in a pseudonymized form to ensure patient confidentiality.

## 3 Results

### 3.1 Sample characteristics

Of the 131 patients, 125 were cis female and 6 were cis male. The average (± SD) age was 25.8 ± 11.2 years, the average length of stay was 26.9 ± 21.7 days, the average BMI was 14 ± 2.6 (kg/m^2^), the average nadir BMI was 13.34 ± 2.47 (kg/m^2^), the average disease duration was 7.67 ± 9.12 years, and 42% were characterized as “binge-purge type” at the time of admission. None of the patients were being treated with prescribed diuretics at admission. **Table 1** displays sociodemographic, anthropometric and laboratory data, as well as concurrent medication, features related to AN and outcomes by group (hyponatremia vs. normonatremia at admission).

**Table 1:** Clinical characteristics, medication use, and laboratory values at admission according to sodium status.

Variable	Total cohort (N = 131)	P-Sodium ≥135 (n=114)	P-Sodium <135 (n=17)	P-value
Clinical characteristics				
Age (years)	25.7 ± 11.2	24.8 ± 10.3	32.2 ± 14.4	0.01*
BMI (kg/m²)	14.0 ± 2.6	14.3 ± 2.5	12.1 ± 2.4	<0.01*
Nadir BMI (kg/m²)	13.3 ± 2.5	13.7 ± 2.4	11.2 ± 2.1	<0.01*
Length of stay (days)	26.8 ± 21.7	25.8 ± 21.2	34.0 ± 24.5	0.14
Disease duration (years)	7.7 ± 9.1	6.7 ± 8.2	13.8 ± 12.6	<0.01*
Female sex, n (%)	125 (95%)	111 (97%)	14 (82%)	0.66
Restrictive subtype, n (%)	73 (57%)	60 (53%)	13 (76.5%)	0.12
Purge subtype, n (%)	56 (43%)	52 (47%)	4 (23.5%)	0.12
Deceased at time of data collection, n (%)	13 (10%)	7 (6%)	6 (35%)	<0.01*
Medication use at admission				
Hormonal contraceptives, n (%)	17 (13.1%)	16 (14.1%)	1 (5.9%)	0.70
Psychiatric comorbidity, n (%)	51 (39.2%)	45 (39.5%)	6 (35.3%)	0.80
SSRI, n (%)	21 (16.2%)	19 (16.8%)	2 (11.8%)	0.99
Antipsychotics, n (%)	40 (30.8%)	35 (30.9%)	5 (29.4%)	0.99
Benzodiazepines, n (%)	11 (8.5%)	10 (8.8%)	1 (5.9%)	0.99
Laboratory values				
Hemoglobin (mmol/L)	7.9 ± 1.2	8.0 ± 1.2	7.5 ± 1.6	0.15
Platelets (10^9^/L)	242 ± 87	234 ± 78	294 ± 125	0.03*
White blood cells (10^9^/L)	5.4 ± 2.6	5.4 ± 2.4	5.5 ± 3.4	0.81
Albumin (g/L)	45.1 ± 5.9	45.8 ± 5.5	40.5 ± 6.7	<0.01*
Potassium (mmol/L)	3.6 ± 0.6	3.6 ± 0.5	3.4 ± 0.9	0.07
Creatinine (µmol/L)	67.1 ± 22.2	65.6 ± 16.3	77.7 ± 43.9	0.03*
Bicarbonate (mmol/L)	25.0 ± 3.7	24.7 ± 3.2	27.4 ± 5.8	0.04*
Sodium (mmol/L)	138.9 ± 4.6	140.2 ± 2.8	129 ± 3.4	<0.01*

Baseline characteristics of the cohort stratified by sodium status. Medication use at admission, and laboratory parameters are presented for the total cohort and stratified by sodium status (P-Sodium ≥135 vs. <135 mmol/L). Values are shown as mean ± SD or n (%). Between-group comparisons were performed using Student’s t-test for continuous variables and χ² test or Fisher’s exact test for categorical variables, as appropriate. P-values represent comparisons between groups and statistical significance is marked with *.

BMI, body mass index; SSRI, selective serotonin reuptake inhibitor; P-, plasma.

At discharge, 15 out of the 17 patients with hyponatremia were discharged with normalized sodium concentrations. Among patients with hyponatremia, mean sodium concentration at discharge was 138.2 ± 4.2 mmol/L, corresponding to a mean increase of Δ 8.5 ± 4.0 mmol/L during admission.

### 3.2 Hyponatremia

**Figure 1:**
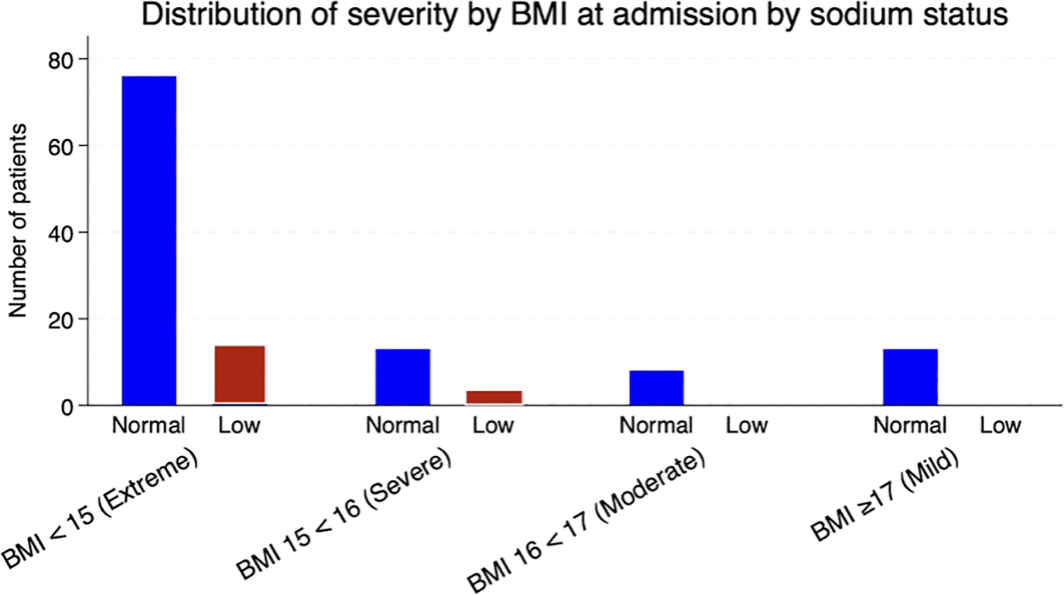
Distribution of BMI severity categories at hospital admission according to sodium status. Bar chart showing the number of patients within DSM-5 BMI severity categories (Extreme: <15 kg/m²; Severe: 15–<16 kg/m²; Moderate: 16–<17 kg/m²; Mild: ≥17 kg/m²), stratified by sodium status (normal vs. hyponatremia) at admission.

At admission, 17 patients had hyponatremia (13%; see **Table 1** for comparison to normonatremia). Comparing patients with normonatriemia to hyponatremia, statistically significant differences were found: The hyponatremia group had a lower BMI (Δ-3.4 kg/m^2^, p<0.01), lower nadir BMI (Δ -4.1 kg/m^2^, p<0.01), longer disease duration (Δ 7.1 years, p<0.01), slightly higher p-creatinine (Δ 12.1 µmol/L, p=0.03), higher platelets (Δ 2.18 10^9^/L, p=0.03), lower P-albumin (Δ -5.3, p<0.01). Using Fisher’s exact test or Chi^2^-test, as appropriate, AN subtype (restricted/purge), psychiatric comorbidities (yes/no), hormonal contraceptives (yes/no), SSRI intake (yes/no), antipsychotics (yes/no), and benzodiazepines (yes/no) all yielded p-values P>0.05, thus, considered non-significant. The distribution of BMI and clinical severity according to DSM-5 criteria and BMI is presented in **Figure 1**.

At discharge, 15 out of the 17 patients with hyponatremia were discharged with normal levels of sodium. For the hyponatremia group, the average sodium levels at discharge were 138.2 ± 4.1 mmol/L, with an increase of Δ 8.5 ± 4.0 mmol/L (P<0.01).

#### 3.2.1 Exploratory analyses according to severity of hyponatremia

In additional exploratory analyses, patients were stratified according to sodium severity at admission (≥135 mmol/L, 130–134 mmol/L, and <130 mmol/L) (See **Table 2**). Progressively higher age, lower BMI, lower nadir BMI, longer disease duration, lower albumin concentrations, and higher creatinine concentrations were observed across worsening sodium categories. Crude mortality rates also increased stepwise from 6.4% among patients with normal sodium levels to 30.0% among patients with sodium concentrations of 130–134 mmol/L and 42.9% among patients with sodium concentrations <130 mmol/L.

**Table 2:** Clinical characteristics on select parameters according to severity of hyponatremia at admission.

Variable	Normal sodium ≥135 mmol/L (n=114)	P-Sodium 130–134 mmol/L (n=10)	P-Sodium <130 mmol/L (n=7)	P-value
Clinical characteristics				
Age (years)	23.9 ± 8.9	30.9 ± 16.7	34.0 ± 11.4	<0.01*
BMI (kg/m²)	14.2 ± 2.5	12.9 ± 2.3	10.9 ± 2.2	<0.01*
Nadir BMI (kg/m²)	13.6 ± 2.3	11.8 ± 2.0	10.4 ± 2.1	<0.001*
Disease duration (years)	6.6 ± 7.6	12.5 ± 14.6	15.9 ± 9.7	<0.01*
Restrictive subtype, n (%)	58 (53.2%)	7 (70.0%)	6 (85.7%)	0.151
Binge-purge subtype, n (%)	51 (46.8%)	3 (30.0%)	1 (14.3%)	0.151
Female sex, n (%)	105 (96.3%)	9 (90.0%)	7 (100%)	0.522
Deceased at follow-up, n (%)	7 (6.4%)	3 (30.0%)	3 (42.9%)	0.002*
Medication use at admission				
SSRI, n (%)	27 (24.8%)	1 (10.0%)	3 (42.9%)	0.272
Antipsychotics, n (%)	32 (29.4%)	1 (10.0%)	3 (42.9%)	0.341
Benzodiazepines, n (%)	12 (11.0%)	1 (10.0%)	0 (0%)	1.000
Laboratory values				
Albumin (g/L)	45.9 ± 5.3	40.9 ± 7.9	40.0 ± 5.0	0.001*
Creatinine (µmol/L)	65.9 ± 16.2	71.5 ± 18.9	86.6 ± 66.6	0.048*
Bicarbonate (mmol/L)	24.7 ± 3.2	29.7 ± 6.1	24.3 ± 2.9	<0.001*

Values are presented as mean ± SD or n (%). Continuous variables were compared using one-way ANOVA and categorical variables using Fisher’s exact test. P-values represent overall comparisons across the three sodium categories.

BMI, body mass index; SSRI, selective serotonin reuptake inhibitor; P-, plasma.

* denotes significant differences in overall comparisons across the three sodium categories (p < 0.05).

### 3.3 Regression models

In unadjusted analyses, reported in **Table 3**, hyponatremia at admission was strongly associated with mortality (OR 8.03, 95% CI 2.29–28.16; p=0.001). However, after adjustment for age, BMI, disease duration, AN subtype, and sex, the association was attenuated and no longer statistically significant (adjusted OR 2.31, 95% CI 0.57–9.42; p=0.24). Increasing age was independently associated with higher mortality risk (adjusted OR per year 1.12, 95% CI 1.03–1.21; p=0.007), whereas BMI, disease duration, and subtype were not significantly associated with mortality in the multivariable model.

**Table 3:** Association between hyponatremia at admission and mortality.

Variable	Unadjusted OR (95% CI)	P-value	Adjusted OR (95% CI)	P-value
Hyponatremia (Low vs. Normal)	8.03 (2.29–28.16)	0.001	2.31 (0.57–9.42)	0.241
Age (per year)	—	—	1.12 (1.03–1.21)	0.007
BMI (per kg/m²)	—	—	0.62 (0.35–1.12)	0.112
Disease duration (per year)	—	—	0.97 (0.89–1.06)	0.512
Restricting subtype (vs. purge)	—	—	0.80 (0.19–3.37)	0.756

Odds ratios (OR) with 95% confidence intervals (CI) from unadjusted and multivariable logistic regression analyses examining the association between hyponatremia (P-Sodium <135 mmol/L) at admission and mortality. The adjusted model included age, BMI at admission, disease duration, AN subtype, and sex. Robust standard errors were applied.

* denotes significant differences in overall comparisons across the three sodium categories (p < 0.05).

## 4 Discussion

### 4.1 Frequency of hyponatremia

This study found a frequency of hyponatremia of 13% for patients admitted to a specialized somatic unit for AN. The results are like the results of earlier studies. A study of 1026 consecutive patients admitted with ED to a recovery center found a prevalence of hyponatremia of 14% (27). Miller et al. reported serious medical findings in outpatients with AN including a low sodium level in 7% of their participants (28). The discrepancy between our data and those of Miller et al. may be attributed to the fact that this study analyzed inpatients (28). Clinical severity has been found to be associated with electrolyte disbalances.

Balling et al. investigated the prevalence of hyponatremia in 2960 hospitalized patients over a broad array of different diseases and found a prevalence of 37% (11). However, a more liberal definition of hyponatremia (<137 mmol/L) was used in their study and patients below 40 years old were excluded (11). Our study population consisted mainly of younger women, with an average age of 25 ± 11.2 years - where hyponatremia is not expected to be highly prevalent.

### 4.2 Associations with hyponatremia

Comparing medical findings in the normonatremic group with the hyponatremic group, significant differences were found for BMI, nadir BMI, age, disease duration, unadjusted mortality, P-albumin, P-bicarbonate, P-platelets, and P-creatinine. The results of our study suggest that hyponatremia is associated with an overall severity of the disease, although, impaired osmoregulation in AN is thought to be multifactorial (16).

The average BMI for the hyponatremic group was 12, with the most severe case having a BMI of 7.6. For perspective, DSM-5 categorizes BMI<15 as extreme severity (12). The average nadir BMI for the hyponatremic group was 11.2. The standardized mortality rate has been calculated to be as high as 45 when nadir BMI is bellow 10.5 (kg/m^2^) (13). The strong association between hyponatremia and mortality observed in unadjusted analyses was markedly attenuated after adjustment for age and markers of illness severity. This pattern suggests that hyponatremia may function primarily as a marker of underlying clinical deterioration rather than an independent causal determinant of mortality in this population. Similar attenuation after multivariable adjustment has been reported in other medical cohorts, where hyponatremia reflects overall disease burden and physiological stress rather than acting as an isolated risk factor (29, 30). In the context of severe AN, hyponatremia may therefore be interpreted as part of a broader phenotype of advanced and chronic medical compromise.

Considering that, the exploratory stratification according to sodium severity also suggested a graded relationship between worsening hyponatremia and markers of illness severity, including lower BMI, lower nadir BMI, longer disease duration, and higher crude mortality. However, the small number of patients with more severe hyponatremia, particularly sodium concentrations <130 mmol/L, limited statistical precision and precluded firm conclusions regarding dose–response relationships with mortality.

Higher P-creatinine and P-bicarbonate levels in the hyponatremic group suggest that renal function may have been affected. Although creatinine values were within the reference range across the cohort, renal impairment cannot be excluded. P-Creatinine is an unreliable marker of kidney function in individuals with severe underweight and reduced muscle mass (31). Despite remaining within normal limits, creatinine was higher in the hyponatremic group, indicating relatively diminished renal function.

Interpretation of renal biomarkers in anorexia nervosa requires caution. Creatinine-based estimates may underestimate renal impairment due to muscle wasting. In this cohort, normal creatinine levels (mean 67.1 ± 22.2 µmol/L) may therefore conceal subclinical renal dysfunction, particularly in patients with prolonged disease duration (mean 7.7 ± 9.1 years).

Impaired osmoregulation in SIADH is known to be caused by certain medications (17). In our study, no significant differences in medication use were found. However, cases of SIADH have been reported in patients with severe AN (16). To correctly diagnose SIADH and differentiate it from reset osmostat syndrome and hypotonic dehydration, measurements of total plasma- and urine-osmolality measurements are required (32). These paraclinical measurements are not routinely assessed in clinical practice today. Recent research has highlighted the need to differentiate between classic SIADH (Type A) and ROS (Type C SIADH) in patients with chronic hyponatremia (33). Unlike classic SIADH, in ROS there is a reduction in the osmotic threshold for ADH release, whereas the tubular capacity of urinary dilution and concentration are preserved. This pattern, with 15 of 17 hyponatremic patients normalizing sodium levels during a relatively short admission, is compatible with a reset osmostat–like phenotype, although this remains speculative in the absence of systematic osmolality and water-loading assessments.

While none of the patients had diagnosed chronic kidney disease (CKD), the observed elevations in plasma creatinine (+12.1 µmol/L, p=0.03) and bicarbonate (+2.7 mmol/L, p=0.01) in the hyponatremic group suggest prerenal mechanisms. These findings likely reflect chronic hypovolemia secondary to malnutrition and dehydration - hallmarks of severe AN. Prerenal azotemia typically presents with elevated blood urea nitrogen (BUN)-to-creatinine ratio (>20:1) and low fractional excretion of sodium (<1%), though these parameters were not routinely assessed.

As previously discussed, hyponatremia in AN is likely multifactorial. In the present study, no significant associations were observed with psychiatric comorbidities, medication use, or purging behavior. Notably, the proportion of patients engaging in purging was lower in the hyponatremic group than in the normonatremic group. Nevertheless, vomiting is typically associated with metabolic alkalosis, and the hyponatremic group did exhibit significantly higher plasma bicarbonate concentrations.

Most patients had received outpatient treatment prior to admission, including oral electrolyte supplementation such as potassium, which may have attenuated more severe electrolyte disturbances (14). Despite this, potassium levels remained at the lower end of the reference range, particularly in the hyponatremic group. These findings should therefore not be extrapolated to patients who do not engage in voluntary outpatient treatment, including those requiring compulsory care. The relative absence of severe electrolyte abnormalities, despite indicators of ongoing purging, may reflect the effectiveness of outpatient harm-reduction strategies.

From a clinical perspective, management of hyponatremia in patients with AN requires careful identification of the underlying mechanism, including hypovolemia, psychogenic polydipsia, SIADH/reset osmostat syndrome, or purging-related electrolyte disturbances. Treatment strategies differ according to pathophysiology but generally involve cautious and gradual correction of sodium levels to avoid osmotic demyelination syndrome, alongside close monitoring of electrolyte concentrations and neurological status (1, 2, 32). In severely malnourished patients, management should additionally include nutritional rehabilitation and targeted interventions addressing excessive fluid intake and eating-disorder behaviors.

### 4.3 Limitations

A strength of this study is the naturalistic clinical population drawn from a highly specialized somatic inpatient unit for severe AN, which enhances the clinical relevance and validity of the findings. The cohort consisted of consecutively admitted patients over a five-year period, reducing selection bias compared with more selected research samples. In addition, patients were clinically well characterized with detailed anthropometric, psychiatric, medication-related, and biochemical data obtained from routine care. By only including the first admission for each patient, we reduced the risk of repeated-measures bias. Finally, the use of both unadjusted and adjusted regression analyses strengthened the interpretation of the observed association between hyponatremia and mortality.

This study also has several limitations. First, the retrospective cross-sectional design precludes causal inference and limits conclusions regarding temporal relationships between hyponatremia and mortality. Second, the relatively small sample size, particularly the low number of deaths and hyponatremic cases, reduces statistical power and increases the risk of imprecise estimates and model instability in multivariable analyses. Third, we lacked systematic measurements of plasma and urine osmolality, urinary sodium, and fractional excretion indices, precluding differentiation between SIADH, reset osmostat syndrome, hypovolemia, or other mechanisms underlying hyponatremia. Fourth, renal function was assessed solely by plasma creatinine, which may underestimate renal impairment in patients with severe muscle wasting. Fifth, mortality data were limited to all-cause death at the time of data collection without detailed information on cause or timing of death, restricting interpretation of pathophysiological links. Finally, as this was a single-center study conducted in a highly specialized somatic inpatient unit for severe AN, generalizability to less severely ill patients or outpatient populations may be limited.

### 4.4 Conclusions

We report a 13% frequency of hyponatremia in patients admitted to a specialized somatic unit for ED. Hyponatremia was associated with an overall severity of disease, and mortality in unadjusted analyses.

## Data Availability

The datasets presented in this article are not readily available due to legal and ethical restrictions under Danish data protection legislation. Access to individual-level health data requires approval from the relevant data protection authorities and cannot be shared without such authorization. Data may be made available from the corresponding author upon reasonable request and with permission from the appropriate regulatory bodies. Requests to access the datasets should be directed to jeanie.meincke.egedal@rsyd.dk.
